# *S*-nitroso- and nitro- proteomes in the olive (*Olea europaea* L.) pollen. Predictive *versus* experimental data by nano-LC-MS

**DOI:** 10.1016/j.dib.2017.09.058

**Published:** 2017-10-06

**Authors:** Rosario Carmona, María José Jimenez-Quesada, Elena Lima-Cabello, José Ángel Traverso, Antonio Jesús Castro, M. Gonzalo Claros, Juan de Dios Alché

**Affiliations:** a Plant Reproductive Biology Laboratory, Department of Biochemistry, Cellular and Molecular Biology of Plants, Estación Experimental del Zaidín (CSIC), Profesor Albareda 1, 18008 Granada, Spain; b Departamento de Biología Molecular y Bioquímica, Universidad de Málaga, Málaga, Spain

**Keywords:** Immunoprecipitation, Proteome, S-nitrosylation, Transcriptome, Tyrosine nitration

## Abstract

The data presented here are related to the research article entitled “Generation of nitric oxide by olive (*Olea europaea* L.) pollen during *in vitro* germination and assessment of the *S*-nitroso- and nitro-proteomes by computational predictive methods” doi:10.1016/j.niox.2017.06.005 (Jimenez-Quesada et al., 2017) [1]. Predicted cysteine *S*-nitrosylation and Tyr-nitration sites in proteins derived from a *de novo* assembled and annotated pollen transcriptome from olive tree (*Olea europaea* L.) were obtained after using well-established predictive tools *in silico*. Predictions were performed using both default and highly restrictive thresholds. Numerous gene products identified with these characteristics are listed here. An experimental validation of the data, consisting in nano-LC-MS (Liquid Chromatography-Mass Spectrometry) determination of olive pollen proteins after immunoprecipitation with antibodies to anti-*S*-nitrosoCys and anti-3-NT (NitroTyrosine) allowed identification of numerous proteins subjected to these two post-translational modifications, which are listed here together with information regarding their cross-presence among the predictions.

**Specifications Table**

**Table:** 

Subject area	*Biology*
More specific subject area	*NO signaling in plant tissues*
Type of data	*Tables*
How data was acquired	*Predictive software, nano-liquid chromatography-mass spectroscopy*
Data format	*Analyzed*
Experimental factors	*Mature pollen (obtained from dehiscent anthers)*
Experimental features	a) *Massive challenge of the predictive tools iSNO-AAPair, GPS-SNO, SNOSite and GPS-YNO2 with a pollen transcriptome (ReprOlive).* b) *Immunoprecipitation of olive pollen proteins with either anti-3-NT polyclonal (Millipore) or anti-S-nitrosoCys polyclonal (Sigma-Aldrich) antibodies, SDS-PAGE, excision of bands and nano-LC-MS analysis.*
Data source location	*Experimentally used samples (mature pollen) collected at Estación Experimental del Zaidín (CSIC), Granada, Spain.*
Data accessibility	*Data are within this article*

**Value of the data**

• Obtained data can be compared with dataset obtained from other sources or predictions, enabling for example comparisons between vegetative/reproductive tissues, diploid/haploid material or signaling capabilities throughout differential metabolic pathways.
• nano-LC-MS determinations allowed building a list of structural, enzymatic, regulatory etc. proteins that should be further assessed regarding whether these gene products are really modified by NO *in vivo*, determining under what conditions they can be modified, the effects of such modifications, reversibility of the modifications (*S*-nitrosylation), and many other aspects.
• Information listed here can be used to improve performance of the predictive tools used here and to create new programs: algorithms are usually modified after feedback from experimental studies. Proteins listed here after MS identification and non-predicted by the *in silico* tools are a good start point for such improvements.

## 1 Data

Predicted *S*-nitrosoproteome of olive pollen is provided through 3 different tables (Table 1–3), which include the use of three different predictive tools and different restrictive conditions for the prediction. Valuable additional information is also included, like Protein id in ReprOlive database, *S*-nitrosylation position/s, Tyrosine nitration position/s, FLN (Full-LengtherNext: an annotation tool) ortholog, FLN status and FLN definition and TAIR database ortholog.

Tables 4–5 provide information regarding predicted Tyr-nitroproteome of olive pollen, which include the use of a predictive tool under two different restrictive conditions for the prediction. Valuable additional information is also included, like Protein id in ReprOlive database, Tyr-nitration position/s, *S*-nitrosylation position/s, FLN ortholog, FLN status and FLN definition and TAIR database ortholog.

Experimentally-determined *S*-nitrosoproteome of olive pollen is provided in the form of a table (Table 6) with information about the peptides identified, annotation details and searches of these proteins throughout the tools for prediction of *S*-nitrosylation and Tyr-nitration.

Experimentally-determined Tyr-nitroproteome of olive pollen is provided in the form of a table (Table 7) with information about the peptides identified, annotation details and searches of these proteins throughout the tools for prediction of *S*-nitrosylation and Tyr-nitration.

## 2 Experimental design, materials and methods

### 2.1 Use of predictive software

For *S*-nitrosylation prediction, freely available tools iSNO-AAPair (http://app.aporc.org/iSNO-AAPair) [2], GPS-SNO (http://sno.biocuckoo.org) [3] and SNOSite (http://csb.cse.yzu.edu.tw/SNOSite/) [4] were challenged with an olive pollen transcriptome described in the ReprOlive database (http://reprolive.eez.csic.es) [5] using different thresholds, when available. For Tyr-nitration prediction, the GPS-YNO2 (http://yno2.biocuckoo.org) [6] tool was used instead, also using two different thresholds. Bioinformatic analysis pipelines were adapted to integrate the use of such bionformatic tools while dealing with big query protein sets [7].

### 2.2 Immunoprecipitation and nano LC-MS analysis

Proteins from 'Picual' mature pollen homogenates were subjected to immunoprecipitation with either an anti-3-NT polyclonal (Millipore) or anti-*S*-nitrosoCys polyclonal (Sigma-Aldrich) antibodies conjugated to protein A agarose beads (Sigma-Aldrich), which were then collected by centrifugation and washed. Samples were subjected to SDS-PAGE gels. Bands of interest were excised after colloidal Coomassie staining of gel, and subjected to nano LC-MS analysis using an NanoAcquity nano-HPLC (Waters), equipped with a Waters BEH C18 nano-column (LC step). Mass spectrometry was performed in a Synapt G2Si ESI Q-Mobility-TOF spectrometer (Waters) equipped with an ion mobility chamber (T-Wave-IMS). Database searching was performed using MASCOT 2.2.07 (Matrixscience, London, UK) against an ad hoc in house made database corresponding to *Olea europaea* L [1], [5].

## Funding sources

This work is part of Ph.D. thesis by María José Jiménez-Quesada, and was supported by European Regional Development Fund (ERDF) co-funded projects BFU2011-22779, BFU2016-77243-P, RTC-2015-4181-2 and RTC-2016-4824-2 (Spanish MINECO), P2011-CVI-7487 (Junta de Andalucía) and 201540E065 (CSIC).

## Footnotes

Appendix A Supplementary data associated with this article can be found in the online version at doi:10.1016/j.dib.2017.09.058.
