# Supplementary material for: S-nitroso- and nitro- proteomes in the olive (Olea europaea L.) pollen. Predictive versus experimental data by nano-LC-MS
Source: Data Brief. 2017 Oct 6;15:474–7. doi: 10.1016/j.dib.2017.09.058 (PMC5647517; doi:10.1016/j.dib.2017.09.058)
Supplement: Supplementary file 1 — Supplementary material [file mmc1.pdf]

GOBIERNO DE ESPAÑA | MINISTERIO DE ECONOMÍA, INDUSTRIA Y COMPETITIVIDAD

CSIC CONSEJO SUPERIOR DE INVESTIGACIONES CIENTÍFICAS eez

ESTACIÓN EXPERIMENTAL DEL ZAIDÍN

AUTHORS DECLARATION

We wish to confirm that there are no known conflicts of interest associated with this publication and there has been no significant financial support for this work that could have influenced its outcome.

We confirm that the manuscript has been read and approved by all named authors and that there are no other persons who satisfied the criteria for authorship but are not listed. We further confirm that the order of authors listed in the manuscript has been approved by all of us.

We confirm that we have given due consideration to the protection of intellectual property associated with this work and that there are no impediments to publication, including the timing of publication, with respect to intellectual property. In so doing we confirm that we have followed the regulations of our institutions concerning intellectual property.

We understand that the Corresponding Author is the sole contact for the Editorial process (including Editorial Manager and direct communications with the office). He/she is responsible for communicating with the other authors about progress, submissions of revisions and final approval of proofs. We confirm that we have provided a current, correct email address which is accessible by the Corresponding Author and which has been configured to accept email from juandedios.alche@eez.csic.es

Signed on behalf of all authors by the corresponding author, as follows:

Date: 13th July 2017

Dr. J.D. Alché

e-mail: juandedios.alche@eez.csic.es
web: www.eez.csic.es http://juandediosalche.wix.com/juande

TEL.: 958 18 16 00 Extensión 215
FAX: 958 18 16 09

Dr. Juan de Dios Alché
Investigador Científico
Dept. Bioquímica, Biología Celular y Molecular de Plantas.
Estación Experimental del Zaidín. CSIC
C/Profesor Albareda 1,
18008 GRANADA ESPAÑA
